# LoQANT: An ImageJ Plugin for Quantifying Nuclear Staining in Immunohistochemistry and Immunofluorescence

**DOI:** 10.3390/ijms262110799

**Published:** 2025-11-06

**Authors:** Katerina Cizkova

**Affiliations:** Department of Histology and Embryology, Faculty of Medicine and Dentistry, Palacky University, 77900 Olomouc, Czech Republic; katerina.cizkova@upol.cz; Tel.: +42-0585632266; Fax: +420-585-632966

**Keywords:** nuclear positivity, immunohistochemistry, immunofluorescence, staining quantification

## Abstract

A large number of regulatory proteins are found in both the cytoplasm and the nucleus. Changes in their nuclear abundance are important for cellular signalling, biological activity, and disease mechanisms. Accurate quantification of nuclear staining is therefore essential in studies of cellular function, therapeutic targeting, drug design, and drug resistance. However, manual scoring is time-consuming, unsuitable for high-throughput applications, and introduces potential bias. As expected, manual scoring by six observers with varying levels of expertise led to highly variable results. Moreover, it was far from achieving good interobserver reliability. To overcome these limitations, LoQANT (Localisation and Quantification of Antigen Nuclear sTaining), an open, freely available ImageJ plugin, was developed for reliable and efficient quantification of nuclear signals. LoQANT is a single cell-based approach to assess the proportion of cells with a positive nuclear signal, independent of cytoplasmic staining, in both immunohistochemically and fluorescently stained samples across various sample types. It also provides semiquantitative and quantitative measurements of nuclear staining intensity. The script, its version for batch analysis, and complete user guide are available at GitHub.

## 1. Introduction

A variety of regulatory proteins, including transcription factors such as nuclear receptors, oncogenes and tumour suppressors, are found in both the cytoplasm and the nucleus of cells. Their nuclear localisation is essential for transmitting signals to the transcriptional machinery and thereby influencing biological activity. Transcription factors can only regulate gene expression when present in the nucleus, whereas their retention in the cytoplasm prevents this function. Changes in nuclear abundance may therefore reflect critical regulatory processes in development, differentiation, and tumour transformation [1]. Dysregulation of nucleocytoplasmic transport has been implicated in various pathological conditions, such as neurodegenerative diseases [2] and carcinogenesis [3]. Accurate quantification of nuclear staining is thus essential for understanding cellular functions, disease mechanisms, and therapeutic targeting, in particular: the identification of prognostic and predictive biomarkers, monitoring treatment response, drug design, and overcoming drug resistance. Therefore, assessment of this is relevant across multiple life science disciplines, including cell biology, biochemistry, histology, pathology, embryology, and pharmacology.

The nuclear/cytoplasmic distribution of proteins of interest can be determined using microscopic techniques. Common techniques include light and fluorescence microscopy. For light microscopy, the protein of interest is usually detected by two-step indirect immunohistochemistry (IHC), where the protein is detected by an unlabelled primary antibody followed by an enzymatically labelled secondary antibody. The gold standard is the use of HRP-labelled secondary antibodies and diaminobenzidine (DAB) for visualisation. For fluorescence microscopy, the target protein can also be detected with antibodies if the secondary one is labelled with fluorophore, or if the target protein can be directly labelled with GFP [4].

However, manual scoring is time consuming and not suitable for high-throughput applications. It also introduces significant intra- and interobserver variability, leading to potential bias [5]. When quantifying nuclear staining, the antigen of interest may be present at varying intensities in both the nucleus and the surrounding cytoplasm. Under these conditions, observers can be influenced by a phenomenon known as simultaneous contrast, where the perception of colour or intensity in one area is influenced by the surrounding context. This effect can cause regions of a slide to appear lighter or darker than the surrounding tissue, leading to inconsistent assessment [6]. Although observer training is not critical to achieving reproducible results, experienced observers tend to perform scoring more quickly [7]. Furthermore, interobserver variability in the interpretation of immunohistochemical staining is also influenced by observer personality traits [8].

Although several plugins for ImageJ/Fiji exist for evaluating immunohistochemical and immunofluorescence staining, none are fully adequate for assessing nuclear localisation of antigens that also exhibit cytoplasmic positivity. IHC Profiler can evaluate DAB staining [9] and offers cytoplasmic and nuclear modes; however, these modes differ only in whether positivity is detected automatically (cytoplasmic mode) or by user-defined thresholds (nuclear mode), and the algorithm does not distinguish subcellular localisation. The output consists of pixel counts for each category of positivity and total field intensity, without providing counts of positive cells, or moreover, the background pixels. Andy’s Algorithms [10], suitable for automated analysis, uses the DAB_IHC macro to measure the count and area of a total selection (haematoxylin and DAB+) and positive selection (DAB+ only), as well as positive intensity. Images are analysed sequentially for total and positive selections, but the macro does not resolve subcellular localisation of the antigen. For fluorescent staining, QuantIF [11], which was originally developed for the detection of viral infections and reports the percentage of cells with nuclear positivity, is used. The macro creates masks of nuclei and masks of signals, and the results are generated by performing an AND operation on the binary images. However, it is not possible to determine the intensity. The aim of this study is to present LoQANT, an open and freely available ImageJ plugin for reliable and efficient quantification of nuclear protein staining, and to demonstrate its applicability by analysing ligand-induced changes in the nuclear localisation of peroxisome proliferator-activated receptor alpha (PPARα) and phosphorylated p38 (phospho-p38) in HT-29 cells.

## 2. Results

### 2.1. Manual Scoring Results

**Figure 1:**
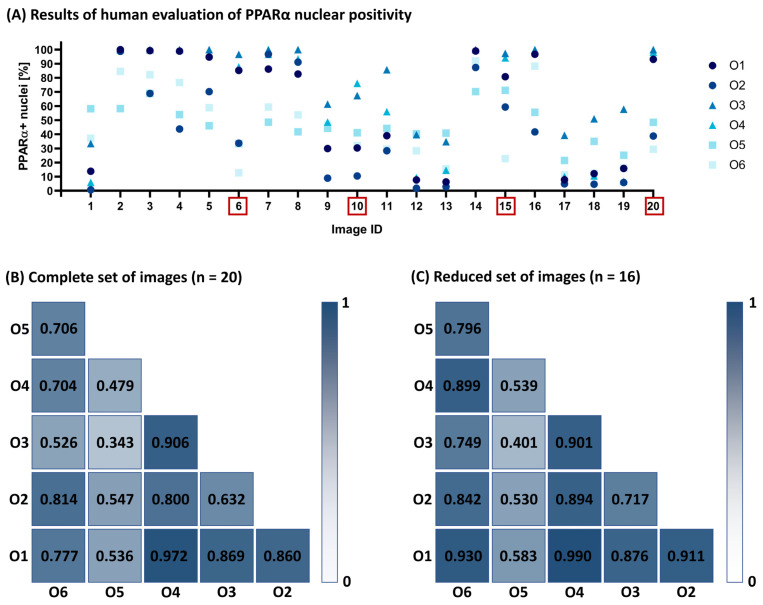
Human evaluation of PPARα nuclear positivity and interobserver reliability. Observers O1 and O2 were experts, O3 and O4 were trained observers, and O5 and O6 were untrained observers. (**A**) Results of human evaluation of nuclear positivity of PPARα presented as percentage of PPARα+ nuclei. The graph shows the values reported by each observer for every image in the complete set of images (20 images). Red rectangles highlight images with >60 percentage point range between observers, which were excluded from the reduced dataset. (**B**) Intraclass correlation coefficients (ICC) for all pairwise observer comparisons. Reliability did not reach the threshold for good agreement (>0.75) in most cases. ICC >0.9 indicates excellent reliability. (**C**) After excluding 4 images with >60 percentage point differences, the reduced set comprised 16 images. ICCs for all pairwise observer comparisons are shown. Observer O5 showed poor-to-moderate agreement and was excluded from further human–programme comparisons.

As expected, manual scoring revealed high interobserver variability, with interobserver differences ranging from 29.8 to 83.8 percentage points for a given image (see Figure 1A). Consequently, the reliability of the scoring was not sufficient to achieve good agreement (coefficients > 0.75) between each pair of observers (see Figure 1B). O5 showed the worst reliability, achieving only poor-to-moderate agreement. The expert observers (O1 and O2) achieved good reliability but not an excellent one (ICC = 0.860). Good reliability was achieved between expert observer O1 and the other observers, with the exception of the previously mentioned O5. Good reliability was also achieved between O2 and O4 and also between O2 and O6. Excellent reliability was achieved between observer O1 and O4 with ICC = 0.972 and O3 and O4 (ICC = 0.906). The order of observers by average ICC from highest to lowest was as follows: O1, O4, O2, O6, O3, and O5. This showed that interobserver agreement was not directly dependent on the level of experience of the observer.

As the results of the manual scoring were used to determine which nuclei were considered positive in LoQANT, the set of images was reduced to 16; images with ranges greater than 60 percentage points were excluded. After reducing images to 16, the ICC value for all users was 0.933. Although all ICC increased, observer O5 still showed poor-to-moderate agreement with most of the others. The coefficients describing the inter-user agreement in the reduced image set are shown in Figure 1C.

To investigate the interobserver variability in more details, each observer’s percentage score against consensus for each image was evaluated (see Supplementary File S1). Although observer O5 did not show the highest mean difference from the consensus (mean diff. = −10.03 for complete set of images and −9.11 for reduced set of images), the variability of their scores was the greatest among all observers, as indicated by the highest standard deviation of the mean differences (SD = 24.91 for complete set of images and SD = 27.13 for reduced sample set). This pattern suggests inconsistent scoring across images. Moreover, O5 reached only moderate agreement with the consensus (ICC = 0.615 for complete set of images and ICC = 0.628 for reduced set of images) and only moderate correlation with consensus (r = 0.640 for complete set of images and r = 0.689 for reduced set of images). Therefore, O5 was excluded from further calibration because good reliability (ICC > 0.75) and strong correlation (r > 0.7) with the consensus were not achieved.

In addition to the interobserver variability, the manual scoring was also time consuming. The average time required for manual scoring for three pre-selected images (the same for each observer) was 12:06, 9:07, and 12:43 min, respectively. The order of observers by average time required for scoring from the fastest to the slowest was as follows: O4, O1, O3, O5, O6, and O2. This showed that the time taken to score did not depend directly on the level of experience of the observer.

### 2.2. LoQANT Description and Testing

**Figure 2:**
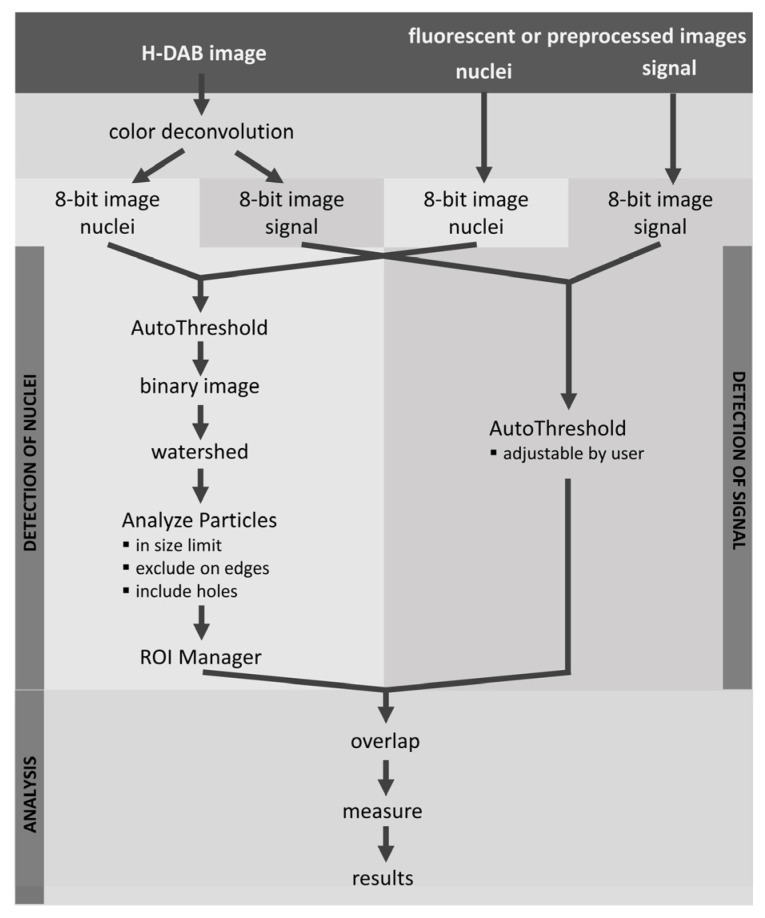
General overview of the algorithm. Flowchart showing the basic steps of the algorithm for evaluating nuclear positivity in H-DAB or fluorescent specimens. Nuclei are first detected, followed by detection of the positive antibody signal. Finally, the two masks are overlapped to quantify nuclear positivity.

**Figure 3:**
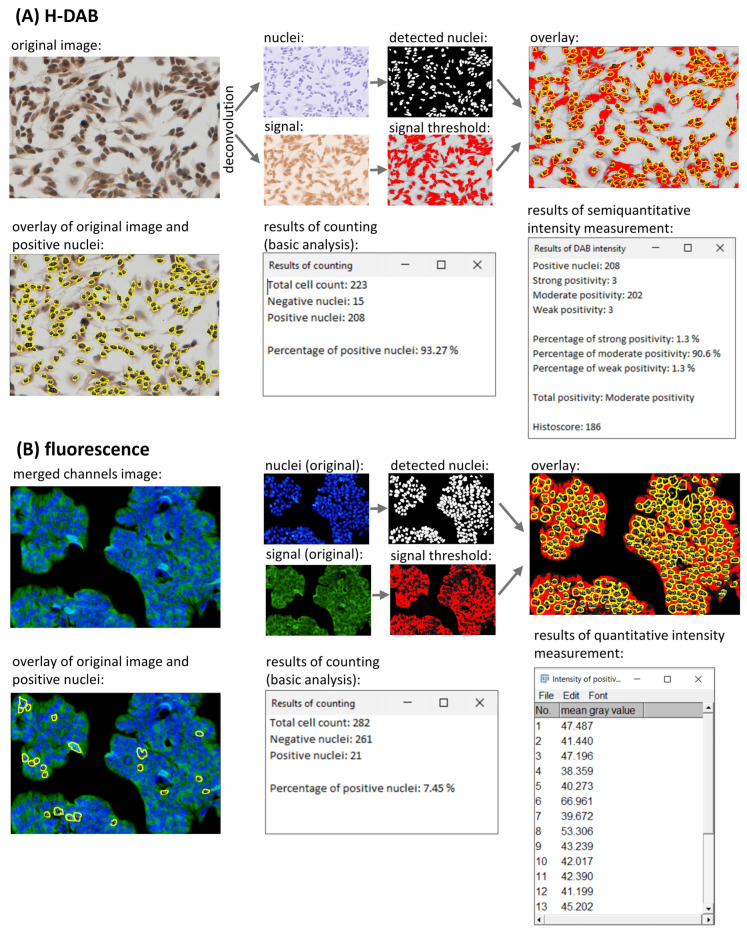
Analysis procedure and results for H-DAB and fluorescence staining. (**A**) H-DAB-stained sample (magnification 200×) analysed using semiquantitative measurement which classifies nuclei into the following intensity levels: negative, weak, moderate, and strong. (**B**) Fluorescence-stained sample (magnification 200×) analysed using quantitative measurement of nuclear signal. The intensity is measured as mean gray value for each measured nuclei (all or only positive nuclei can be measured) and the results are provided in the results table. Intensity measurement is optional and can be applied as needed.

LoQANT script together with user manual and images for testing are freely available at https://github.com/Keri-histo/LoQANT (accessed on 6 January 2025). LoQANT is a single cell-based approach designed to assess the percentage of cells with nuclear positivity in an image written in the Python 2.7 programming language. It evaluates nuclear positivity based on the positive nuclear area. The analysis consists of the following steps: (1) automatic detection of nuclei, (2) automatic identification of antibody staining (with user-modifiable settings), and (3) determination of positive nuclei by overlapping regions. The fraction of positive signal within each nucleus is calculated and the percentage of cells with nuclear positivity is reported. A general overview of the algorithm is shown in Figure 2. The results of analysis are shown in Figure 3.

The input depends on the staining method. The basic methods are “H-DAB” and “fluorescence”. LoQANT accepts one RGB image for H-DAB staining and two RGB or grayscale images for the fluorescence method. The next step is to set the 8-bit image for nuclei and antibody signal. For H-DAB, the images are automatically adjusted after colour deconvolution. For fluorescence, images are assigned by user. If any preprocessing on deconvoluted images prior LoQANT analysis is needed, “preprocessed images: H-DAB” method can be used. For this option, the script accepts two images: one for nuclei and one for signal. The appropriate images are also set by the user in the following dialogue and the following steps in the analysis are the same as for H-DAB or fluorescence.

In the next step the nuclei are detected using the built-in Analyze Particles function of ImageJ/Fiji version 2.14.0/1.54f. The size of the detected objects is limited by the user in the initial dialogue. The nuclei are detected automatically according to the selected thresholding algorithm in initial dialogue. All AutoThreshold algorithms available in ImageJ/Fiji are available for thresholding: Default, Huang [12], Huang2, Intermodes [13], IsoData [14], Li [15], MaxEntropy [16], Mean [17], MinError [18], Minimum [13], Moments [19], Otsu [20], Percentile [21], RenyiEntropy [16], Shanbhag [22], Triangle [23], and Yen [24]. All detected objects are added to the ROI manager and the area is measured. The antibody signal is then detected according to the selected thresholding algorithm in initial dialogue. In contrast to the detection of nuclei, the user can change the value using a slider. The nuclei areas stored in the ROI manager are then displayed on the signal image and the threshold area is measured. The fraction of positive signal in each nucleus is then determined and the percentage of cells with a positive nucleus is calculated.

**Figure 4:**
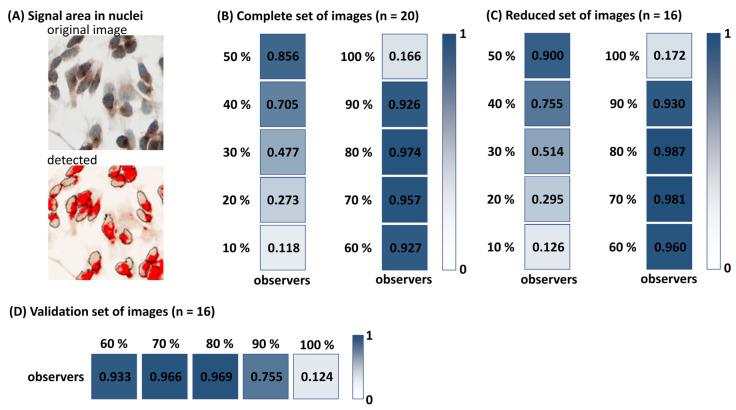
Optimisation of threshold for defining nuclear positivity and its validation. (**A**) Example showing that positive signal typically covers less than 100% of the nuclear area, magnification 200×(**B**,**C**) Human observer scoring was used to determine the proportion of nuclear area that must be covered by the signal to classify nuclei as positive. Reliability between human observers (O1, O2, O3, O4, O6) and LoQANT scoring at thresholds ranging from 10% to 100% of positive nuclear area was assessed using intraclass correlation coefficients (ICC). The highest agreement was obtained at the 80% threshold for both full and reduced image sets. This threshold was therefore set as the default for LoQANT, although it can be adjusted by the user. (**D**) Validation of the 80% threshold. Reliability between human observers (O1, O2, O3, O4) and LoQANT scoring at thresholds ranging from 60% to 100% of positive nuclear area for validation. The set of images (n = 15) was assessed using intraclass correlation coefficients (ICC).

LoQANT evaluates the positivity of the nuclei based on the positive nuclear area. As the positive signal does not usually represent 100% of the nucleus area, data from human observers were used to determine how much of the nucleus area must be covered by the signal for the nuclei to be considered positive. The highest reliability between LoQANT and human observers was achieved when the positive signal area represented 80% of the nucleus. The ICC between human observers (mean value for O1, O2, O3, O4, O6) and the tested positive area fraction are shown in Figure 4. The default value in LoQANT is 80%, but this can be changed by the user in the initial dialogue.

LoQANT was tested on 9473 cells for H-DAB and 8388 cells for fluorescence. While initially tested on cells grown directly on slides, LoQANT was also successfully applied to various sample types, including cell smears and both physiological and tumour tissue samples (see Supplementary Files S2). The average processing time per image was 7 s on a laptop with an AMD Ryzen 3 4300U processor, or 11 s when intensity measurement was included, significantly reducing the time required for analysis compared to manual scoring (over 11 min on average). LoQANT has been successfully tested with Fiji version 2.14.0/1.54f on Windows 10, Windows 11, macOS Monterey 12.5., and Ubuntu 22.04 LTS operating systems. LoQANT is also available in a version for batch processing.

### 2.3. Intensity Measurement

In addition, LoQANT offers both semiquantitative and quantitative measurements of nuclear staining intensity. The semiquantitative approach is recommended for H-DAB staining. Semiquantitative analysis classifies nuclei into three intensity levels: weak, moderate, and strong [25,26]. The classification is based on mean gray value thresholds [9]: strongly positive, mean gray value < 60; moderately positive, mean gray value between 60 and 120; and weakly positive, mean gray value ≥ 120. In addition, a 1% cut-off is applied: if ≥99% of the nuclei are negative, the sample is automatically classified as “negative”. The analysis also determines the histoscore (H-score) and an overall positivity category for each field of view [25,26]. The histoscore has been widely used in pathology research and has potential applications in the evaluation of predictive biomarkers [27]. The histoscore is calculated as follows [5]: histoscore = (% strongly positive × 3) + (% moderately positive × 2) + (% weakly positive × 1). The overall intensity score is calculated as follows [9]: score = (% strongly positive × 4) + (% moderately positive × 3) + (% weakly positive × 2) + (% negative × 1). Based on the calculated score, an overall intensity category is assigned: scores > 301 indicate strong positivity, scores between 201 and 301 indicate moderate positivity, and scores between 101 and 201 indicate weak positivity.

Unlike enzymatic DAB staining, fluorescence-based detection is fully quantifiable. For quantitative intensity measurement, the user can choose to measure the intensity of all detected nuclei or only those nuclei classified as positive. The mean gray value is returned for each case, allowing detailed intensity analysis. The results of intensity measurement are shown in Figure 3.

### 2.4. Validation of the Threshold for Nuclear Positivity

The 80% nuclear area coverage threshold used by LoQANT to classify nuclei as positive was validated using a validation set of images. The 80% threshold yielded the highest agreement with the human consensus (mean of observers for each image), showing the highest ICC (ICC = 0.969; excellent reliability) and Pearson correlation coefficient (r = 0.965; very strong correlation). However, the differences in ICC and correlation were very small compared with the 60% and 70% thresholds, indicating overall robustness of the method within this range. ICCs are provided in Figure 4D, detailed results are provided as Supplementary File S3.

### 2.5. Incorporation of AI-Based Segmentation Approaches to the Workflow

The number of nuclei detected per image by StarDist, Trainable WEKA, and human observers was compared to assess segmentation accuracy. StarDist counts showed excellent agreement and very strong correlation with human counts (ICC = 0.976, r = 0.943) with a mean difference of +1 nuclei per image, indicating minimal systematic bias. Trainable WEKA showed similar performance (ICC = 0.956, r = 0.915) with a mean difference of −7 nuclei per image. Overlay images further confirmed visually accurate delineation of nuclear boundaries. These validation results support the reliability of AI-based segmentation. For results see Supplementary File S4.

To evaluate the impact of different segmentation strategies on LoQANT performance, nuclear positivity was quantified using the “preprocessed images: H-DAB” method of LoQANT with masks generated by StarDist and Trainable WEKA. The results obtained with both segmentation approaches were nearly identical, demonstrating excellent agreement with ICC = 0.9988, indicating an almost perfect consistency between the two methods. For results see Supplementary File S5.

### 2.6. Analysis of Nuclear Positivity of PPARα and p-p38 Using LoQANT

LoQANT provided rapid analysis of nuclear positivity of PPARα and p-p38. Using batch version of LoQANT, the analysis was finished in less than 4 min per antigen. The analysis was performed using 20 images per treatment group, with the following number of cells per image: H-DAB staining for PPARα, 171 ± 63; fluorescent staining for PPARα, 245 ± 66; and H-DAB staining for phospho-p38, 246 ± 109.

The analysis revealed a significant increase in the percentage of cells with nuclear PPARα positivity after treatment with fenofibrate and WY-14643 compared to controls (*p* < 0.05), consistent with ligand-induced nuclear translocation of PPARα. In contrast, treatment with the antagonist GW6471 significantly decreased the percentage of nuclear-positive cells relative to controls (*p* < 0.05), in agreement with its expected inhibitory effect. This effect was demonstrated in both H-DAB and fluorescent samples. Evaluation using histoscore showed a significant increase in nuclear PPARα only after treatment with the activators fenofibrate and WY-14643, whereas GW6471 did not induce a statistically significant change (*p* = 0.2264). Analysis of fluorescence intensity revealed a significant change after fenofibrate and GW6471 treatment but not after WY-14643 treatment (*p* = 0.6806).

**Figure 5:**
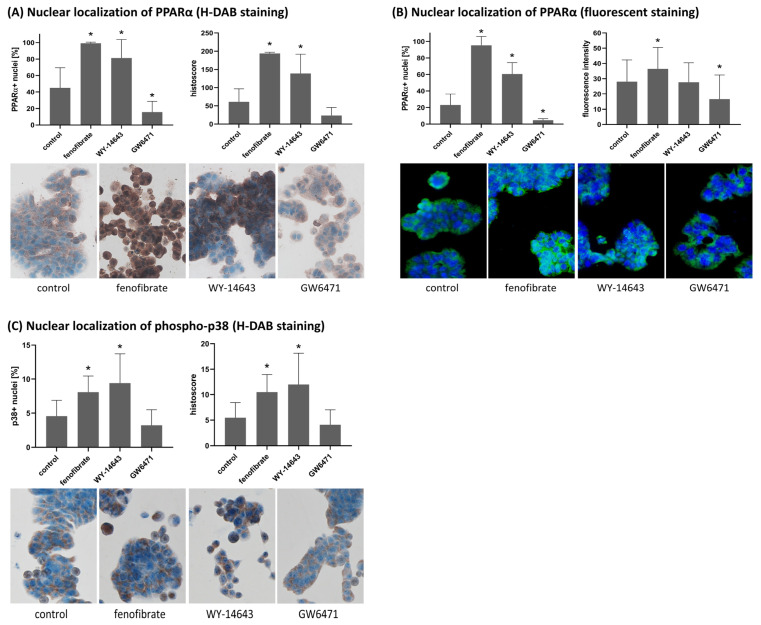
LoQANT analysis of nuclear positivity for PPARα and phospho-p38 following treatment with PPARα modulators. (**A**) Percentage of PPARα-positive nuclei and histoscore values in H-DAB-stained HT-29 cells. (**B**) Percentage of PPARα-positive nuclei and nuclear fluorescence intensity (mean gray value) measured in HT-29 cells with fluorescence-based detection. Note that results for the percentage of positive nuclei are consistent across both staining methods. (**C**) Percentage of phospho-p38-positive nuclei in H-DAB-stained HT-29 cells. In all cases, HT-29 cells were treated with fenofibrate (150 µM), WY-14643 (200 µM), or GW6471 (10 µM) as indicated. Data represent mean ± SD from 20 fields of vision per group. Asterisk (*) indicates a statistically significant difference compared to the control group (*p* < 0.05, Student’s *t*-test). Magnification 200×.

The same experimental design was applied to assess the subcellular distribution of phosphorylated p38, a stress-activated kinase known to translocate to the nucleus upon activation. The LoQANT-based quantification showed a significant increase in the percentage of cells with nuclear phosphor-p38 positivity after treatment with fenofibrate and WY-14643 (*p* < 0.05), whereas GW6471 treatment produced no significant change compared to controls. The histoscore evaluation showed the same results. These findings indicate that PPARα activation may be associated with enhanced phospho-p38 activation and nuclear localisation in HT29 cells. For results see Figure 5. The significant difference obtained for phosphor p38 nuclear localisation was relatively low. To ensure adequate statistical power, sample size calculations in G*Power (α = 0.05, power = 0.8) using the observed standard deviations were performed. The required sample sizes for detecting the observed effects of fenofibrate and WY-14643 were 6 images per group (control and fenofibrate), 17 images per group (control and WY-14643) for percentage of positive nuclei, and 17 images per group for histoscore analysis. Therefore, the use of 20 images per treatment group provides sufficient power to detect significant differences, supporting the reliability of the observed effects.

## 3. Discussion

Nucleocytoplasmic protein shuttling is an important regulatory mechanism in cell biology and disease. While changes in nuclear signal can reflect such processes, accurate quantification of nuclear staining itself is critical for understanding cellular function and disease mechanisms, as well as for therapeutic targeting, drug design, and overcoming drug resistance. The aim of this study was to evaluate the interobserver variability in assessing the percentage of cells with positive nuclear staining and to provide a standardised ImageJ-based approach for this type of analysis in DAB- or immunofluorescence-stained samples.

As expected, manual scoring produced highly variable results. It was far from achieving good reliability between any two observers. Even the expert observers achieved good but not excellent reliability. Interobserver variability is a well-known phenomenon in manual scoring. The previous study performed by Jaraj et al. showed that subjective assessment of intensity can be performed with a high level of reproducibility, while estimation of staining extent is less reliable [7]. The other study performed by Varghese et al. comparing the scores of three pathologists showed that the scores may vary significantly [9]. Our study also showed that observer agreement, as well as time expenditure, is not dependent on the level of experience of the observers. These findings are partially consistent with a previous study that concluded that level of training was not critical for obtaining reproducible results, although experienced observers were faster [7]. Interestingly, interobserver variability in assessment of immunohistochemical staining is also influenced by observer personality [8]. Pathologists with high conscientiousness scores had the lowest interobserver variability, the highest diagnostic accuracy, and reported fewer tumours as positive. In contrast, those with high neuroticism scores had higher variability, lower accuracy, and reported more tumours as positive [8]. All of the above demonstrates the need for standardisation.

LoQANT evaluates the positivity of the nuclei based on the positive nuclear area. The 80% nuclear area coverage threshold was identified as the optimal criterion for classifying nuclear positivity in LoQANT, yielding the highest concordance with human consensus. It is acknowledged, however, that this validation was conducted using the same cell line and antigen. Extension of the evaluation to independent cohorts and biologically diverse tissue types is therefore recommended to further substantiate the generalizability of this parameter. Importantly, LoQANT allows users to modify the nuclear area coverage threshold according to their experimental needs. For low-abundance nuclear antigens or weakly expressed targets, applying a lower threshold may provide a more accurate representation of nuclear positivity, as the default 80% cut-off could lead to underestimation. Users are thus encouraged to empirically determine and validate the most appropriate threshold for their specific datasets.

A major advantage of LoQANT is that it exclusively evaluates the positive signal in the nucleus, independent of cytoplasmic staining. To the best of the author’s knowledge there is currently no straightforward way to perform this type of dedicated nuclear positivity analysis in ImageJ/Fiji. The essential step for successful LoQANT analysis is accurate detection of nuclei. By default, LoQANT employs the AutoThreshold method provided by ImageJ/Fiji to segment nuclei and signals. However, in some cases this approach may not provide optimal results. To address this limitation, LoQANT provides an option to analyse preprocessed images (“preprocessed images: H-DAB” method) allowing segmentation of nuclei by external tools prior to analysis. This feature supports flexible integration of different segmentation strategies, including AI-based approaches available for ImageJ such as StarDist [28], Trainable WEKA [29], or CellPose [30]. In the present study, this functionality is illustrated by comparing nuclear masks generated with StarDist (deep learning) and Trainable WEKA (classical machine learning), followed by analysis in LoQANT when both approaches produced highly consistent results.

LoQANT can be used across a wide range of sample types. It has been successfully applied to a variety of biological images, including cells grown directly on slides, smears, and formalin-fixed, paraffin-embedded tissue samples. Other advantages of the provided algorithm include elimination of interobserver visual perception bias and requiring minimal supervision for analysis; thus, it significantly eliminates the dependence on a trained pathologist/histologist, reducing the time burden for analysis, requiring only a few steps to follow the analysis and measurement of staining intensity.

As an option, LoQANT provides measurement of staining intensity using two methods: semiquantitative and quantitative. The semiquantitative measurement provides the number of positive nuclei in three discrete categories: weak, moderate, and strong intensity [25]. The determination of the category is based on the mean gray value using the previously described category threshold [9]. In addition, the histoscore and overall positivity category for a given field of view are also determined [25,26]. This semiquantitative approach is used for H-DAB-stained specimens. DAB staining is not stochiometric, therefore the darkness of the stain does not equate to the expression of a particular antigen. It does not follow the Beer–Lambert law, which describes a linear relationship between the concentration of a compound and its absorbance or optical density. As a result, dark-stained DAB has a different spectral shape than light-stained DAB. In addition, H-DAB staining also uses a series of amplification steps to visualise the results [31,32,33]. Although IHC is a qualitative laboratory assay and the extent of linearity of the assay is unknown, some linearity is assumed; lower staining intensity is expected to represent weaker expression of the antigen of interest and vice versa, at least in most clinically used IHC biomarker assays. The histoscore has been widely used in pathology research and has also been shown to be potentially useful for some predictive biomarkers [27]. In contrast to enzymatic detection by DAB, fluorescence is fully quantifiable and LoQANT provides the option to measure intensity of each nucleus.

The biological examples included in this study demonstrate the practical usefulness of LoQANT in quantifying nuclear staining of PPARα and phospho-p38 in colorectal adenocarcinoma-derived HT-29 cells. Both proteins are known to undergo nucleocytoplasmic shuttling, and changes in their nuclear abundance are functionally relevant. PPARα is a nuclear receptor that regulates genes involved in lipid metabolism, energy homeostasis, and inflammation. In HT29 cells, fenofibrate and WY-14643 increased nuclear PPARα, whereas GW6471 decreased it, consistent with their expected effects [34,35,36,37]. p38 is a stress-activated kinase that regulates gene expression, cell cycle checkpoints, and apoptosis. Given its critical role in colorectal cancer progression and therapy resistance, targeting the nuclear translocation or activity of p38α emerges as a promising therapeutic strategy. In colorectal cancer, nuclear p38α activity has been linked to chemoresistance, whereas its inhibition can sensitise cancer cells to chemotherapeutic agents such as 5-fluorouracil. Moreover, blocking nuclear p38 has been shown to inhibit tumour growth in inflammation-associated colon cancer models and to improve responses to chemotherapy [38,39,40]. LoQANT analysis showed that nuclear phospho-p38 increased after fenofibrate and WY-14643 treatment but was unaffected by GW6471, suggesting a link between PPARα activation and p38 nuclear translocation. This highlights a potential link between PPARα activation and p38 nuclear translocation [41,42] which should be confirmed in follow-up studies or orthogonal assays. These results emphasise that automated, high-throughput quantification of nuclear positivity can reveal subtle yet biologically significant changes in protein localisation. LoQANT is therefore a powerful tool for quantifying nuclear staining, providing objective and reproducible measurements. By enabling precise and reliable assessment of nuclear signals, the plugin complements traditional manual methods and can support studies of dynamic protein localisation, including processes involving nucleocytoplasmic shuttling.

When using LoQANT, it is recommended that good image analysis practices are followed to obtain valuable results, e.g., fixation, staining, image acquisition settings, exposure, contrast, and brightness are consistent across a cohort of samples [43]. For LoQANT to work properly, the size of the nuclei in pixels must be specified. This will filter out artefacts or overlapping nuclei. Furthermore, it is recommended to test which of the available algorithms for automatic thresholding is suitable for the measured samples.

LoQANT is a dedicated ImageJ/Fiji plugin designed for the targeted quantification of nuclear protein localisation. Other open-source platforms for image analysis, such as QuPath [44] and CellProfiler [45], are also available, in addition to Fiji. Both QuPath and CellProfiler are general-purpose image analysis tools. QuPath is optimised primarily for whole-slide and tissue-level studies, providing advanced algorithms for cell detection and intensity-based classification. It can quantify nuclear staining intensity in both H-DAB and fluorescent images, and compute parameters such as the percentage of positive cells, H-score and Allred score. However, batch analysis in QuPath is usually carried out using user-customised scripts [44]. CellProfiler, on the other hand, enables flexible, high-throughput image quantification through modular, customisable pipelines, but it requires extensive parameter optimisation and is not specifically optimised for the assessment of nuclear localisation [45]. By contrast, LoQANT offers a lightweight, task-specific tool that is seamlessly integrated within the ImageJ/Fiji environment. This facilitates robust and user-friendly quantification of nuclear positivity.

In summary, LoQANT is a computational image processing tool that provides an unbiased and reproducible evaluation of nuclear staining. It assesses the positive signal in the nucleus, independent of cytoplasmic staining, in both H-DAB- and fluorescence-stained samples across multiple sample types. LoQANT significantly reduces analysis time and eliminates the need for highly trained observers, offering a robust and efficient alternative to traditional manual scoring methods.

## 4. Material and Methods

### 4.1. Cell Samples

SH-SY5Y and HT-29 cell lines were obtained from the American Type Culture Collection (ATCC Numbers: CRL-2266 and HTB-38, respectively) and authenticated by STR profiles prior to the experiment by the Department of Clinical Genetics, Palacky University, Olomouc. Cells were routinely cultured in DMEM (HT-29; Sigma Aldrich, St. Louis, MI, USA, Cat. No. D6171) and DMEM/F12 (SH-SY5Y; Sigma Aldrich, D8437) supplemented with 10% FBS (HyClone, Marlborough, MA, USA, Cat. No. SV30160.03), penicillin (100 U/mL), and streptomycin (100 mg/L). Cells were incubated at 37 °C in 5% CO_2_ and passaged twice a week.

Cells were seeded on 8-well cell culture slides (SPL Life Sciences, Gyeonggi-do, Republic of Korea, Cat. No. 30108) at a density of 18,000 cells/well, adhered overnight and treated with the following PPARα ligands: 150 µM fenofibrate (Cayman Chemicals, Ann Arbor, MI, USA, Cat. No. 10005368), 200 µM WY-14643 (Sigma-Aldrich, Cat. No. C7081), or 10 µM GW6471 (Cayman Chemicals, Cat. No. 11697) to achieve a broad spectrum of nucleocytoplasmic distribution of the antigen of interest. The cells were incubated with ligands for 72 h. Stock solutions of PPARα ligands were prepared by dissolving in DMSO. The control cells were treated by an appropriate concentration of DMSO (0.5%).

After incubation period, the cells were fixed in 4% paraformaldehyde for 10 min in RT and stained for the nuclear receptor PPARα (GeneTex, San Antonio, TX, USA, Cat. No. GTX28934, dilution 1:200) and phospho-p38α (dilution 1:1000, Invitrogen, Waltham, MA, USA, Cat. No. MA-5-15177). For H-DAB and fluorescent staining method, the slides were rehydrated, cell membranes were permeabilized with 0.01% Triton-X100, and heat-induced antigen retrieval was performed in citrate buffer pH 6 for phosphor-p38α or EDTA pH9 for PPARα. In both cases, this step was performed at 120 °C for 15 min in Histos device. After pre-treatment with PolyDetector Peroxidase Blocker (Bio SB, Santa Barbara, CA, USA part of the detection kit) for 5 min and ProteinBlock (Dako, Glostrup, Denmark) for 30 min, samples were incubated with primary antibodies for 1 h at RT. For H-DAB method, visualisation was performed using the Mouse/Rabbit PolyDetector DAB HRP Brown Kit (Bio SB, Santa Barbara, CA, USA, Cat. No. BSB 0205). For fluorescent method, Opal520 fluorophore from Opal 3-Plex Manual Detection Kit (Akoya bioscience, Marlborough, MA, USANEL810001KT) was used according to vendor protocol. Briefly, the slides were incubated with Opal Anti-Ms + Rb HRP for 10 min (part of the kit) and then with Opal520 for 10 min. Finally, heat-induced antigen retrieval was performed again. The nuclei were counterstained with haematoxylin or DAPI depending on the method of visualisation and cover slipped. Tris buffer with TWEEN 20 (pH 7.6) was used for washing between different steps.

Images were captured using an Olympus BX40 microscope equipped with an Olympus DP71 camera (Olympus, Shinjuku, Japan) at 200× magnification. All images were taken at a resolution of 1920 × 1200 pixels and saved as .tif files. Twenty images with different intensity of immunostaining in the nucleus and cytoplasm of each method were selected for interobserver variability testing and LoQANT settings. Moreover, the validation set of different images (n = 15) of SH-SY5Y cells stained for PPARα was collected.

### 4.2. Manual Samples Evaluation of PPARα

The selected H-DAB-stained images were manually scored by six observers with different levels of experience: two experts (O1 and O2), two trained observers (O3 and O4), and two observers with no previous experience (O5 and O6). Both O5 and O6 underwent identical training provided by the same expert shortly before the analysis which included scoring of different images under supervision. A session was conducted prior to the main analysis where observers received identical instructions and reference examples of nuclear positivity. All of them used the manual counting tool provided in ImageJ/Fiji. The evaluation resulted in the percentage of cells with nuclear positivity for each image. In addition, the observers were asked to measure the time taken to score three pre-selected images. The reliability between each pair of observers was evaluated using the intraclass correlation coefficient (ICC). Moreover, each observer’s percentage score against consensus defined as the mean of all other observers for each image was evaluated. Mean difference, standard deviation (SD), 95% confidence interval (CI) of the mean difference, intraclass correlation coefficient (ICC), and Pearson correlation coefficient (r) between each observer and the consensus were calculated.

### 4.3. Agreement Between Observers and LoQANT

The results obtained from the observers were used to determine which nuclei would be classified as positive by LoQANT. The same image set was analysed with LoQANT with varying portions of positive nuclei in the range from 0.1 to 1.0, with 0.1 increments. The nuclei and signal segmentation algorithms were set to “Default”. The size of nuclei was limited to 300–1200 based on pre-measurement areas of 50 cells of the cell line tested (may vary for other cell lines). The measured results (% of positive nuclei) for each overlap fraction were compared with the user results. For each image, the human consensus defined as mean % of cells with positive nuclei were calculated from the values obtained by observers O1, O2, O3, O4, and O6. Observer O5 was excluded (see Section 2.1). Then, the reliability (ICC) between human observers and LoQANT was calculated.

### 4.4. Validation of the 80% Threshold for Nuclear Positivity

To validate the 80% nuclear area coverage threshold used to define nuclear positivity, a validation set of 15 different images was evaluated by observers O1, O2, O3, and O4 (observers O5 and O6 were unavailable). The same validation sample set was then processed in LoQANT using nuclear coverage thresholds of 60%, 70%, 80%, 90%, and 100%. The size of the nuclei was limited to 300–1200, the nuclei and signal segmentation algorithm were set to “Default” (as described previously). The reliability (ICC) between human consensus (mean of human observers for each image) and LoQANT was calculated. Moreover, mean difference, standard deviation (SD), 95% confidence interval (CI) of the mean difference, and Pearson correlation coefficient (r) between human consensus and LoQANT were also evaluated.

### 4.5. Incorporation of Existing AI-Based Segmentation Fiji Plugins to the Analysis Workflow

AI-based plugins for Fiji can be readily integrated into the LoQANT workflow by supplying pre-segmented images as input through the “preprocessed images: H-DAB” option of LoQANT. To demonstrate this, nuclear segmentation of the same set of 20 H-DAB-stained SH-SY5Y cell images was performed using the StarDist 2D [28] and Trainable WEKA Segmentation [29] plugins. For deep learning-based segmentation, nuclei were detected using StarDist with the pretrained 2D “versatile (H&E nuclei)” model on original images with default StarDist settings (probability/score threshold = 0.5, overlap threshold = 0.4). These settings were surprisingly very accurate for the tested sample set (see Results section). Prior to classical machine learning-based segmentation, all images were processed with Color Deconvolution available in Fiji. For this approach, Trainable WEKA Segmentation was trained on representative subsets of nuclei and background from the deconvoluted images. The resulting classifier was saved and applied to the entire dataset. The nuclei masks were obtained from both segmentation approaches. After segmentation, the complete set of images was analysed using LoQANT. The input for analysis were the nuclei masks and corresponding DAB signal images.

To validate the AI-based segmentation, the number of nuclei detected per image by StarDist, Trainable WEKA, and human consensus (mean of all observers O1–O4) was compared using mean difference, standard deviation (SD), 95% confidence interval (CI) of the mean difference, intraclass correlation coefficient (ICC), and Pearson correlation coefficient (r). In addition, visual inspection was performed to assess the accuracy of nuclear boundary delineation across the tested sample set.

### 4.6. Application Examples of the Nuclear Positivity Analysis Using LoQANT

To illustrate the biological applicability of LoQANT, the nuclear staining of two proteins that are known to undergo nucleocytoplasmic shuttling, PPARα, and phospho-p38 in HT29 cells was evaluated. The cells were treated and stained using H-DAB and fluorescence method as mentioned above. Images were captured using an Olympus BX40 microscope equipped with an Olympus DP71 camera at 200× magnification. All images were taken at a resolution of 1920 × 1200 pixels and saved as .tif files. Automated analysis with LoQANT was performed at 20 fields of vision per group using the batch version of the script with default settings. In all cases, the % of positive cells were detected. Moreover, the histoscore for H-DAB staining and intensity measurement for immunofluorescence were also used (intensity measurement method: “quantitative: all nuclei (rec for fluorescence)”). The nuclear localisation of PPARα and p-p38 in comparison to control was assessed using Student’s *t*-test (two-tailed).

### 4.7. Statistic Evaluation

Reliability between each pair of observers, between observers and LoQANT, and between LoQANT results after incorporating StarDist and Trainable WEKA Segmentation to the workflow was assessed as intraclass correlation coefficient (ICC), random mixed model, and absolute agreement using IBM SPSS Statistics (ver. 29.0.1.0). Values greater than 0.75 indicate good reliability, while values greater than 0.9 indicate excellent reliability [46]. Moreover, additional parameters such as mean difference, standard deviation (SD), 95% confidence interval (CI) of the mean difference, and Pearson correlation coefficient (r) between each observer and consensus were calculated. A Pearson r greater than 0.7 indicates strong correlation, while values greater than 0.9 indicate very strong correlation [47]. The nuclear localisation of PPARα and p-p38 in comparison to control was assessed using Student’s *t*-test (two-tailed). The sample size required to achieve a statistical power of 80% (α = 0.05) based on the observed standard deviations was calculated using G*Power software (version 3.1.9.7). Graphs were created by GraphPad Prism 8 software.

## Data Availability

Data is contained within the article or Supplementary Material.

## Supplementary Materials

The supporting information can be downloaded at: https://www.mdpi.com/article/10.3390/ijms262110799/s1.

## References

[1] Fu X., Liang C., Li F., Wang L., Wu X., Lu A., Xiao G., Zhang G. (2018). The Rules and Functions of Nucleocytoplasmic Shuttling Proteins. Int. J. Mol. Sci..

[2] Ding B., Sepehrimanesh M. (2021). Nucleocytoplasmic Transport: Regulatory Mechanisms and the Implications in Neurodegeneration. Int. J. Mol. Sci..

[3] Conforti F., Wang Y., Rodriguez J.A., Alberobello A.T., Zhang Y.W., Giaccone G. (2015). Molecular Pathways: Anticancer Activity by Inhibition of Nucleocytoplasmic Shuttling. Clin. Cancer Res..

[4] Molenaar C., Weeks K.L. (2018). Nucleocytoplasmic shuttling: The ins and outs of quantitative imaging. Clin. Exp. Pharmacol. Physiol..

[5] Aeffner F., Wilson K., Martin N.T., Black J.C., Hendriks C.L.L., Bolon B., Rudmann D.G., Gianani R., Koegler S.R., Krueger J. (2017). The Gold Standard Paradox in Digital Image Analysis: Manual Versus Automated Scoring as Ground Truth. Arch. Pathol. Lab. Med..

[6] Paizs M., Engelhardt J.I., Siklós L. (2009). Quantitative assessment of relative changes of immunohistochemical staining by light microscopy in specified anatomical regions. J. Microsc..

[7] Jaraj S.J., Camparo P., Boyle H., Germain F., Nilsson B., Petersson F., Egevad L. (2009). Intra- and interobserver reproducibility of interpretation of immunohistochemical stains of prostate cancer. Virchows. Arch..

[8] Butter R., Hondelink L.M., van Elswijk L., Blaauwgeers J.L.G., Bloemena E., Britstra R., Bulkmans N., van Gulik A.L., Monkhorst K., de Rooij M.J. (2022). The impact of a pathologist’s personality on the interobserver variability and diagnostic accuracy of predictive PD-L1 immunohistochemistry in lung cancer. Lung Cancer.

[9] Varghese F., Bukhari A.B., Malhotra R., De A. (2014). IHC Profiler: An open source plugin for the quantitative evaluation and automated scoring of immunohistochemistry images of human tissue samples. PLoS ONE.

[10] Law A.M.K., Yin J.X.M., Castillo L., Young A.I.J., Piggin C., Rogers S., Caldon C.E., Burgess A., Millar E.K.A., O’Toole S.A. (2017). Andy’s Algorithms: New automated digital image analysis pipelines for FIJI. Sci. Rep..

[11] Handala L., Fiore T., Rouillé Y., Helle F. (2019). QuantIF: An ImageJ Macro to Automatically Determine the Percentage of Infected Cells after Immunofluorescence. Viruses.

[12] Huang L.K., Wang M.J.J. (1995). Image thresholding by minimizing the measures of fuzziness. Pattern Recognit..

[13] Prewitt J.M., Mendelsohn M.L. (1966). The analysis of cell images. Ann. N. Y. Acad. Sci..

[14] Ridler T.W., Calvard S. (1978). Picture Thresholding Using an Iterative Selection Method. IEEE Trans. Syst. Man Cybern..

[15] Li C.H., Tam P.K.S. (1998). An iterative algorithm for minimum cross entropy thresholding. Pattern Recognit. Lett..

[16] Kapur J.N., Sahoo P.K., Wong A.K.C. (1985). A new method for gray-level picture thresholding using the entropy of the histogram. Comput. Vis. Graph. Image Process..

[17] Glasbey C.A. (1993). An Analysis of Histogram-Based Thresholding Algorithms. CVGIP Graph. Models Image Process..

[18] Kittler J., Illingworth J. (1986). Minimum error thresholding. Pattern Recognit..

[19] Tsai W.-H. (1985). Moment-preserving thresolding: A new approach. Comput. Vis. Graph. Image Process..

[20] Otsu N. (1979). A Threshold Selection Method from Gray-Level Histograms. IEEE Trans. Syst. Man Cybern..

[21] Doyle W. (1962). Operations Useful for Similarity-Invariant Pattern Recognition. J. ACM.

[22] Shanbhag A.G. (1994). Utilization of Information Measure as a Means of Image Thresholding. CVGIP Graph. Models Image Process..

[23] Zack G.W., Rogers W.E., Latt S.A. (1977). Automatic measurement of sister chromatid exchange frequency. J. Histochem. Cytochem..

[24] Jui-Cheng Y., Fu-Juay C., Shyang C. (1995). A new criterion for automatic multilevel thresholding. IEEE Trans. Image Process..

[25] Fedchenko N., Reifenrath J. (2014). Different approaches for interpretation and reporting of immunohistochemistry analysis results in the bone tissue—A review. Diagn. Pathol..

[26] Price P., Ganugapati U., Gatalica Z., Kakadekar A., Macpherson J., Quenneville L., Rees H., Slodkowska E., Suresh J., Yu D. (2023). Reinventing Nuclear Histo-score Utilizing Inherent Morphologic Cutoffs: Blue-brown Color H-score (BBC-HS). Appl. Immunohistochem. Mol. Morphol. AIMM.

[27] Avilés-Salas A., Muñiz-Hernández S., Maldonado-Martínez H.A., Chanona-Vilchis J.G., Ramírez-Tirado L.A., Hernández-Pedro N., Dorantes-Heredia R., Ruí Z.M.J.M., Motola-Kuba D., Arrieta O. (2017). Reproducibility of the EGFR immunohistochemistry scores for tumor samples from patients with advanced non-small cell lung cancer. Oncol. Lett..

[28] Schmidt U., Weigert M., Broaddus C., Myers G. (2018). Cell Detection with Star-Convex Polygons. Proceedings of the Medical Image Computing and Computer Assisted Intervention—MICCAI 2018.

[29] Arganda-Carreras I., Kaynig V., Rueden C., Eliceiri K.W., Schindelin J., Cardona A., Sebastian Seung H. (2017). Trainable Weka Segmentation: A machine learning tool for microscopy pixel classification. Bioinformatics.

[30] Stringer C., Wang T., Michaelos M., Pachitariu M. (2021). Cellpose: A generalist algorithm for cellular segmentation. Nat. Methods.

[31] Ruifrok A.C., Johnston D.A. (2001). Quantification of histochemical staining by color deconvolution. Anal. Quant. Cytol. Histol..

[32] Landini G., Martinelli G., Piccinini F. (2020). Colour deconvolution: Stain unmixing in histological imaging. Bioinformatics.

[33] van der Loos C.M. (2008). Multiple immunoenzyme staining: Methods and visualizations for the observation with spectral imaging. J. Histochem. Cytochem..

[34] Tan Y., Wang M., Yang K., Chi T., Liao Z., Wei P. (2021). PPAR-α Modulators as Current and Potential Cancer Treatments. Front. Oncol..

[35] Cizkova K., Foltynkova T., Hanyk J., Kamencak Z., Tauber Z. (2021). When Activator and Inhibitor of PPARα Do the Same: Consequence for Differentiation of Human Intestinal Cells. Biomedicines.

[36] Umemoto T., Fujiki Y. (2012). Ligand-dependent nucleo-cytoplasmic shuttling of peroxisome proliferator-activated receptors, PPARalpha and PPARgamma. Genes Cells.

[37] Xu H.E., Stanley T.B., Montana V.G., Lambert M.H., Shearer B.G., Cobb J.E., McKee D.D., Galardi C.M., Plunket K.D., Nolte R.T. (2002). Structural basis for antagonist-mediated recruitment of nuclear co-repressors by PPARα. Nature.

[38] Maik-Rachline G., Zehorai E., Hanoch T., Blenis J., Seger R. (2018). The nuclear translocation of the kinases p38 and JNK promotes inflammation-induced cancer. Sci. Signal.

[39] Chiacchiera F., Matrone A., Ferrari E., Ingravallo G., Lo Sasso G., Murzilli S., Petruzzelli M., Salvatore L., Moschetta A., Simone C. (2009). p38α blockade inhibits colorectal cancer growth in vivo by inducing a switch from HIF1α- to FoxO-dependent transcription. Cell Death Differ..

[40] Han Z., Meng L., Huang X., Tan J., Liu W., Chen W., Zou Y., Cai Y., Huang S., Chen A. (2022). Inhibition of p38 MAPK increases the sensitivity of 5-fluorouracil-resistant SW480 human colon cancer cells to noscapine. Oncol. Lett..

[41] Phan T., Zhang X.H., Rosen S., Melstrom L.G. (2023). P38 kinase in gastrointestinal cancers. Cancer Gene Ther..

[42] Cizkova K., Tauber Z. (2023). Fibrates Affect Levels of Phosphorylated p38 in Intestinal Cells in a Differentiation-Dependent Manner. Int. J. Mol. Sci..

[43] Cromey D.W. (2010). Avoiding twisted pixels: Ethical guidelines for the appropriate use and manipulation of scientific digital images. Sci. Eng. Ethics..

[44] Bankhead P., Loughrey M.B., Fernández J.A., Dombrowski Y., McArt D.G., Dunne P.D., McQuaid S., Gray R.T., Murray L.J., Coleman H.G. (2017). QuPath: Open source software for digital pathology image analysis. Sci. Rep..

[45] Stirling D.R., Swain-Bowden M.J., Lucas A.M., Carpenter A.E., Cimini B.A., Goodman A. (2021). CellProfiler 4: Improvements in speed, utility and usability. BMC Bioinform..

[46] Koo T.K., Li M.Y. (2016). A Guideline of Selecting and Reporting Intraclass Correlation Coefficients for Reliability Research. J. Chiropr. Med..

[47] Schober P., Boer C., Schwarte L.A. (2018). Correlation Coefficients: Appropriate Use and Interpretation. Anesth. Analg..

