# Supplementary figures and images for: LoQANT: An ImageJ Plugin for Quantifying Nuclear Staining in Immunohistochemistry and Immunofluorescence
Source: Int J Mol Sci. 2025 Nov 6;26(21):10799. doi: 10.3390/ijms262110799 (PMC12610205; doi:10.3390/ijms262110799)

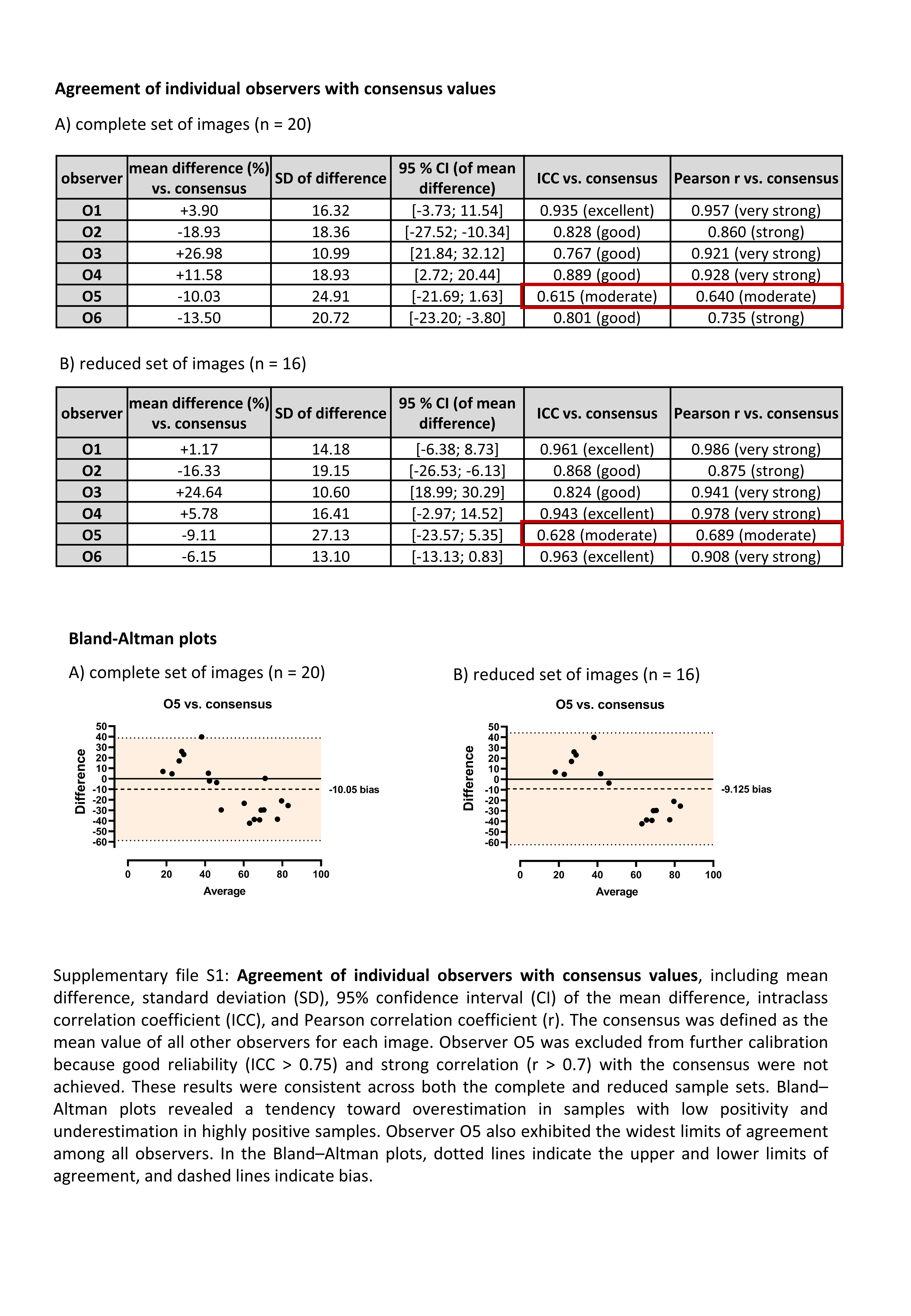

Supplement: Supplementary file 1 [file ijms-26-10799-s001.zip › Supplementary file S1.tif]

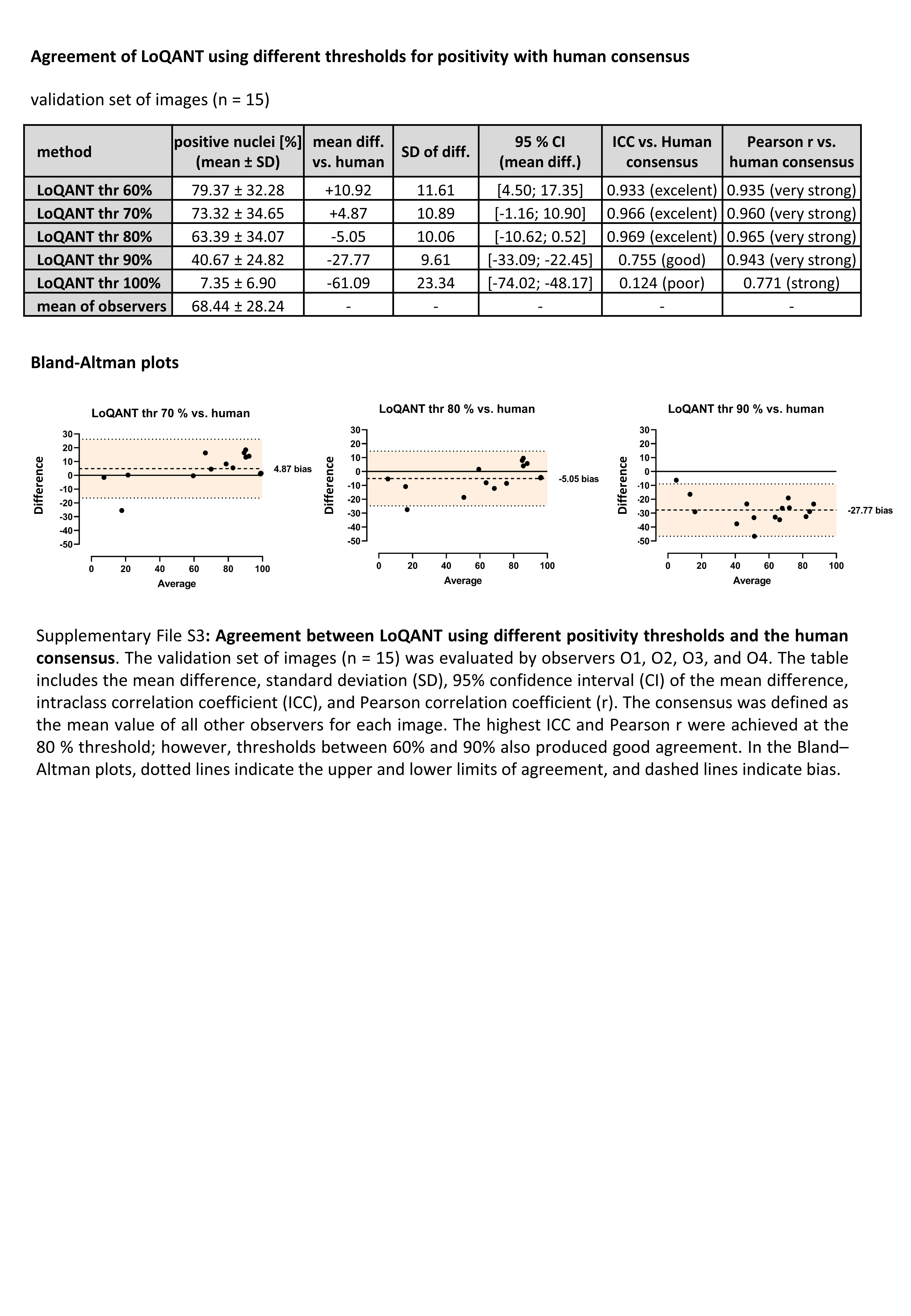

Supplement: Supplementary file 1 [file ijms-26-10799-s001.zip › Supplementary file S3.tif]

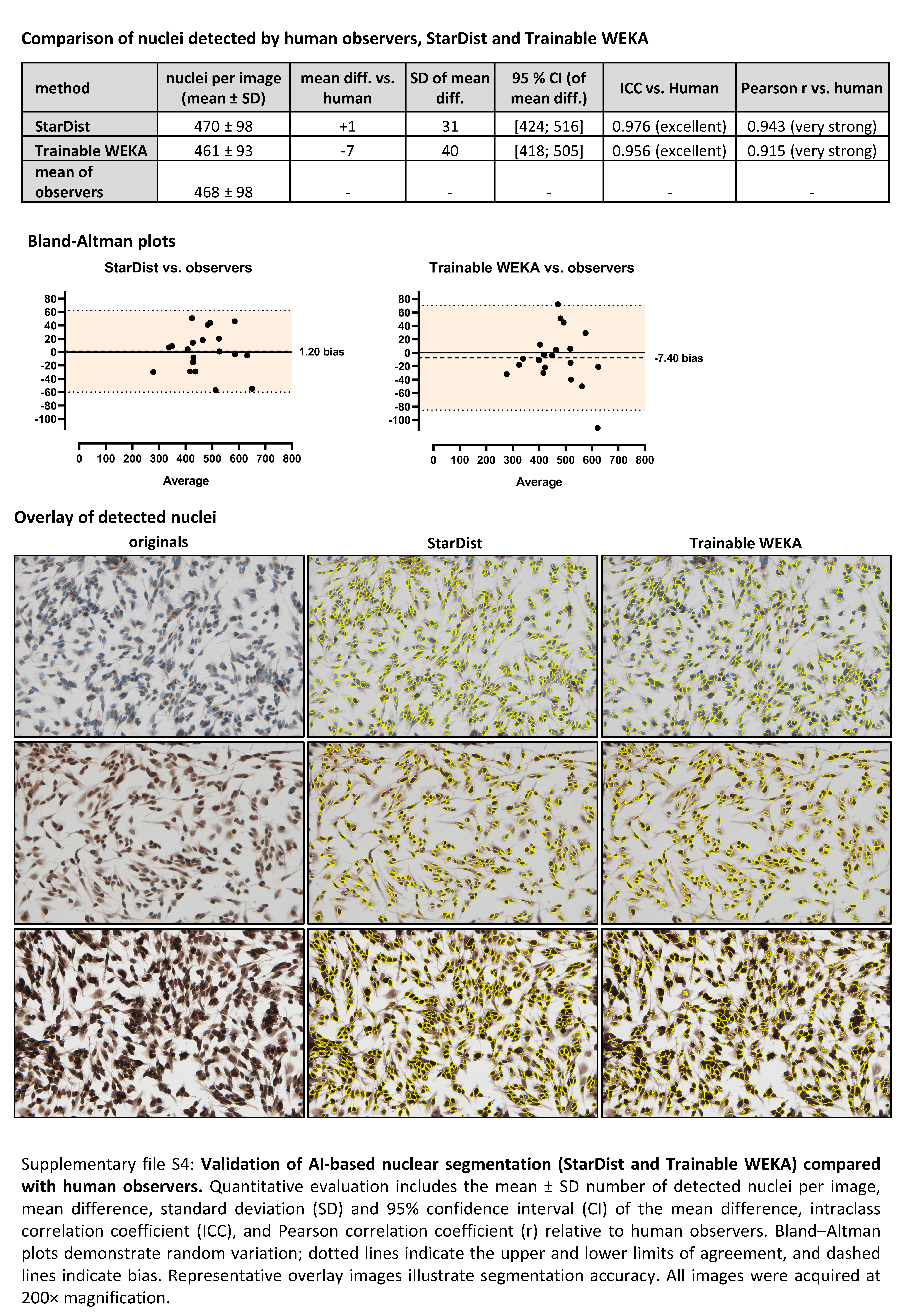

Supplement: Supplementary file 1 [file ijms-26-10799-s001.zip › Supplementary file S4.tif]
